# Trueness and Precision of Intraoral Scanners for 3D-Printed Orthodontic Models with Attachments: An In Vitro Comparative Study

**DOI:** 10.3390/bioengineering13060709

**Published:** 2026-06-20

**Authors:** Fırat Oğuz, Handan Göze Oğuz, Sabahattin Bor

**Affiliations:** 1 Department of Orthodontics, Faculty of Dentistry, İnönü University, Malatya 44280, Türkiye; venaroshan@gmail.com; 2 MEF Dental Oral and Dental Health Clinic, Malatya 44280, Türkiye; aydin_handan_01@hotmail.com

**Keywords:** digital orthodontics, intraoral scanner, 3D-printing, orthodontic models, attachments

## Abstract

**Background:** Advances in additive manufacturing and CAD/CAM technologies have expanded the use of 3D-printed orthodontic models in digital aligner workflows. Intraoral scanners (IOS) are critical for accurately capturing attachment geometries and dental morphology during these workflows. However, comparative evidence regarding IOS accuracy in models with complex orthodontic structures remains limited. Therefore, this study aimed to compare the trueness and precision of five IOS using 3D-printed orthodontic models with attachments. **Methods:** In this in vitro study, thirty independent single-arch 3D-printed models (either maxillary or mandibular) with orthodontic attachments were scanned twice with each IOS. The Smart Optics Vinyl laboratory scanner served as the reference scanner. Scans were aligned and superimposed in CloudCompare, and root mean square (RMS) deviation values were calculated to evaluate accuracy. Nonparametric Kruskal–Wallis and Dunn tests were applied (α = 0.05). **Results:** Significant differences were found among scanners for both trueness and precision (*p* < 0.001). Primescan, TRIOS 3, and iTero element 5D demonstrated comparable trueness (*p* > 0.05) and outperformed Rapideye MI-1000 (*p* < 0.001). iTero element 2 plus showed slightly lower accuracy but remained clinically acceptable. Primescan achieved the highest precision, significantly exceeding iTero element 2 plus, iTero element 5D, and Rapideye MI-1000 (*p* < 0.01). TRIOS 3 also exhibited excellent repeatability, comparable to Primescan (*p* = 1.000). **Conclusions:** All intraoral scanners, except Rapideye MI-1000, demonstrated accuracy levels generally considered clinically acceptable for digital orthodontic and additive manufacturing workflows. Primescan, TRIOS 3, and iTero element 5D exhibited similarly high trueness, while Primescan showed the most consistent precision. The ability of these scanners to reproduce fine anatomical details may improve the reliability of 3D-printed orthodontic models and in-office aligner production workflows.

## 1. Introduction

In recent years, continuous advancements in digital technologies such as computer-aided design and manufacturing (CAD/CAM) systems, additive manufacturing (AM), three-dimensional (3D) scanning, and the printing of advanced dental biomaterials have significantly transformed the field of dentistry, enabling increasingly accurate and predictable digital workflows across a wide range of clinical applications [1,2]. In particular, the increasing integration of 3D printing into orthodontic workflows has enabled the widespread use of in-office aligner production and digitally fabricated orthodontic appliances. Among these innovations, intraoral scanners (IOS) have emerged as a particularly important development because of their ability to enhance digital workflow efficiency, facilitate accurate data acquisition, and support the fabrication of high-fidelity 3D-printed orthodontic models.

IOS systems offer significant advantages over conventional impression techniques for both clinicians and patients. The use of IOS reduces the cost of impression materials, allows for immediate visual control, and significantly decreases the risk of operator-related errors [3,4,5,6]. Digital data transfer saves time, shortens the procedure, and enhances patient comfort. While conventional impression methods can be highly uncomfortable for patients with a strong gag reflex [5,7], digital scanning eliminates this issue. Considering these factors, the adoption of intraoral scanners (IOS) in clinical practice has increased rapidly. Accordingly, the number of studies involving IOS has also grown, and the accuracy of these scanners has become a widely investigated topic in dentistry [1,8,9,10].

In contemporary digital orthodontic workflows, inaccuracies occurring during intraoral scanning may propagate throughout subsequent manufacturing stages, including STL generation, 3D printing, and thermoforming procedures. Therefore, scanner accuracy plays a critical role in ensuring the dimensional fidelity of digitally manufactured orthodontic appliances.

When IOS scans are compared with conventional dental models in terms of trueness, it has been reported that the quality of tissue mapping is comparable to or even superior to that of traditional methods [9,11]. With the increasing adoption of IOS systems, digital scans are no longer limited to restorative or orthodontic applications. They have also enabled new areas of use, including identification of individuals through palatal rugae patterns in forensic science [12], digital determination of occlusal contact points [11], and more precise visualization of interdental areas in patients with periodontal problems [13].

The growing use of 3D-printed orthodontic models and polymer-based aligner systems has further increased the importance of highly accurate digital impressions in additive manufacturing-based orthodontic workflows. IOS technology is particularly prominent in orthodontics due to its wide range of clinical applications [14,15]. IOS is especially critical during the refinement stages of clear aligner therapy. When tooth movement is inadequate or patient compliance is suboptimal, updated intraoral scans allow for precise adjustments to the treatment plan and the fabrication of new aligners with accurately placed attachments. At this point, it is essential that the sharp edges of intraoral attachments are captured with high precision, as deviations introduced during intraoral scanning may propagate through subsequent stages of the digital workflow. Therefore, attachment-bearing models were specifically selected to evaluate the scanners’ ability to reproduce complex geometries relevant to additive manufacturing-based aligner workflows.

Although several studies have investigated the accuracy of different IOS devices, their operating software continues to undergo regular updates, and limited evidence exists regarding their performance in digitally manufactured orthodontic models featuring complex attachment geometries. Furthermore, the influence of scanner accuracy on additive manufacturing-based aligner workflows and the fidelity of 3D-printed orthodontic models remains insufficiently explored. In light of this, the present study aimed to evaluate the trueness and precision of five different IOS on 3D-printed orthodontic models with attachments used in additive manufacturing-based aligner workflows, using Smart Optics as the reference device. The null hypothesis stated that there would be no statistically significant difference (1) in trueness among the five evaluated IOS, and (2) in precision among all evaluated scanners, including the reference scanner.

## 2. Materials and Methods

Ethical approval for the study was obtained from the Non-Interventional Clinical Research Ethics Committee of İnönü University, with the approval number 2025/7144.

An a priori power analysis (G*Power v3.1, Düsseldorf University, Düsseldorf, Germany) for repeated measures ANOVA with a medium effect size (f = 0.25), α = 0.05, and 90% power indicated that at least 26 samples were needed [16]. In the present study, a total of 30 independent single-arch models (15 maxillary and 15 mandibular models) obtained from patients undergoing in-house aligner production were used, ensuring sufficient statistical power and minimizing the risk of Type II error. Although the sample size calculation was based on a parametric repeated-measures design, the final statistical analysis was performed using non-parametric tests because the assumptions of normality and homogeneity of variance were not satisfied after data collection.

### 2.1. 3D Model Printing

The orthodontic models used in this study were produced using a 3D-printing workflow for in-house aligner production. The maxillary and mandibular models, which contained various composite attachment geometries and different aligner attachment configurations, were obtained from patients undergoing digital aligner treatment planning. The models were digitally designed and prepared using Autolign 3D orthodontic software (Diorco Co., Ltd., Yongin-si, Gyeonggi-do, Republic of Korea). After completion of the digital treatment planning, the finalized digital models were exported as STL files for 3D printing.

All models were printed using the Ackuretta SOL LCD 3D printer (Ackuretta Technologies, Taipei, Taiwan) with eSUN orthodontic model resin (Shenzhen Esun Industrial Co., Ltd., Shenzhen, China). Prior to printing, the STL files were prepared in the slicing software according to the manufacturer’s recommended orthodontic printing parameters. Support structures were automatically generated, and the models were positioned to optimize dimensional stability and attachment detail reproduction during printing.

Following printing, all models underwent standardized post-processing procedures. Initially, uncured resin residues were removed using 99% isopropyl alcohol in an Ackuretta Cleani ultrasonic cleaning unit (Ackuretta Technologies, Taipei, Taiwan) with a two-stage cleaning protocol consisting of 5 min followed by an additional 5 min. Subsequently, the models were post-cured for 10 min using an Ackuretta Curie curing unit (Ackuretta Technologies, Taipei, Taiwan) to enhance polymerization stability and surface consistency before scanning. These standardized manufacturing and post-processing procedures were applied to minimize dimensional distortion and ensure reproducibility throughout the digital orthodontic workflow.

### 2.2. Scanning

All models were scanned using a standardized protocol. The scanning sequence began at the upper left-most distal molar, continued occlusally across the full arch, pivoted to the palatal side to capture the palatal surfaces, and then returned along the buccal surfaces in a continuous and systematic manner [8]. A laboratory-type Vinyl scanner (smart optics Sensortechnik GmbH, Bochum, Germany) was used to obtain the reference datasets. Each model was scanned two times per device. Deviation values from superimposed scans were recorded in Microsoft Excel (Excel 365; Microsoft Corp., Redmond, WA, USA) for analysis.

**Figure 1:**
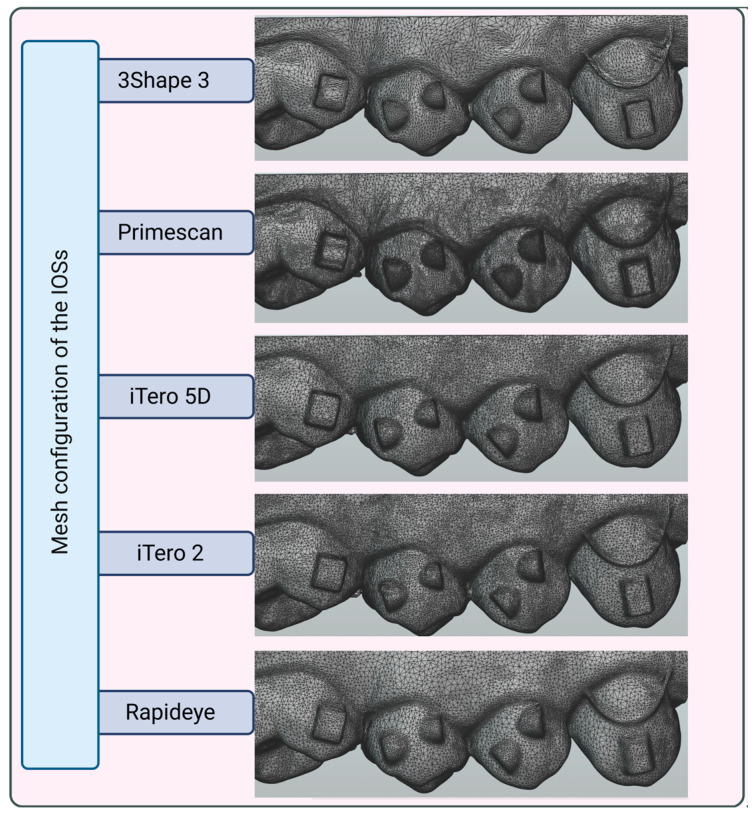
Mesh configuration of STL models scanned by different intraoral scanners.

**Figure 2:**
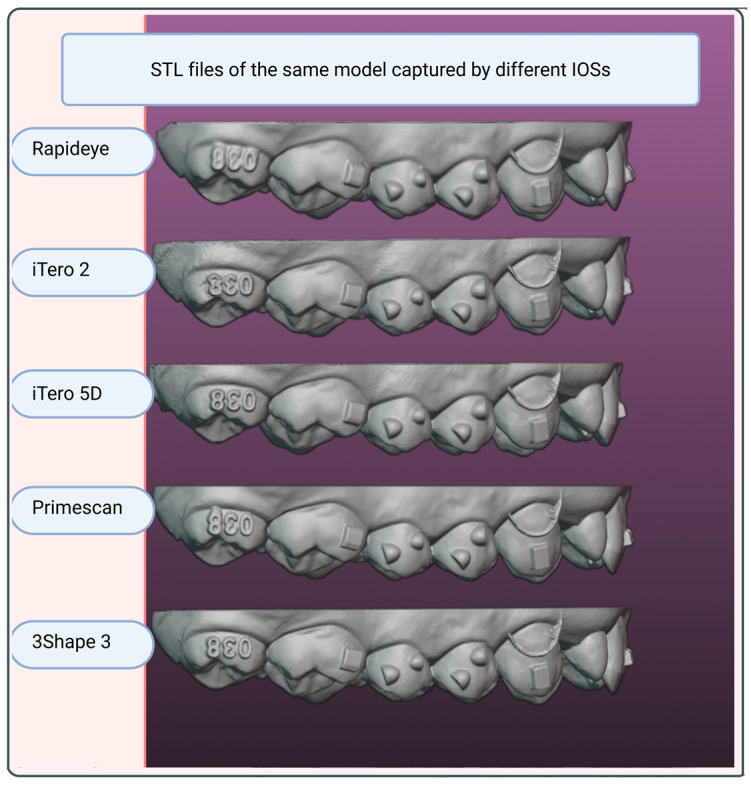
STL surface images of the same model acquired using different intraoral scanners.

The scanners tested were 3Shape TRIOS 3 (3Shape, Copenhagen, Denmark), Rapideye MI-1000 (Mode Medical, Istanbul, Turkey), iTero Element 2 plus (Align Technology, San Jose, CA, USA), and iTero D5 (Align Technology, San Jose, CA, USA) (Table 1). The mesh configuration and STL surfaces of the same model obtained by different intraoral scanners are illustrated in Figure 1 and Figure 2, respectively, to demonstrate differences in data density and anatomical detail reproduction.

### 2.3. Accuracy Assessment

Superimposition was performed using CloudCompare software (Open-Source Project, version 2.12.4, Paris, France). Initially, the models were aligned using at least four corresponding points, followed by fine registration. According to ISO 5725-1 [17], accuracy is defined in terms of trueness and precision. Trueness refers to how close the obtained measurement is to the actual dimensions of the object, whereas precision describes the consistency of repeated measurements of the same object. Root mean square (RMS) values were used to assess trueness and precision.

Root Mean Square (RMS): Calculated using the formula:

RMS = √{(1/n) Σ_i=1_^n^ [(x_i_,test − x_i_,ref)^2^ + (y_i_,test − y_i_,ref)^2^ + (z_i_,test − z_i_,ref)^2^]}.

where x_i_,test, y_i_,test, and z_i_,test denote the coordinates of the i-th point on the test scan; x_i_,ref, y_i_,ref, and z_i_,ref denote the coordinates of the corresponding point on the reference scan; and n is the total number of corresponding point pairs. RMS values were expressed in millimeters (mm).

Denote the coordinates of corresponding points on the test and reference scans, respectively, and *n* is the total number of point pairs.

**Figure 3:**
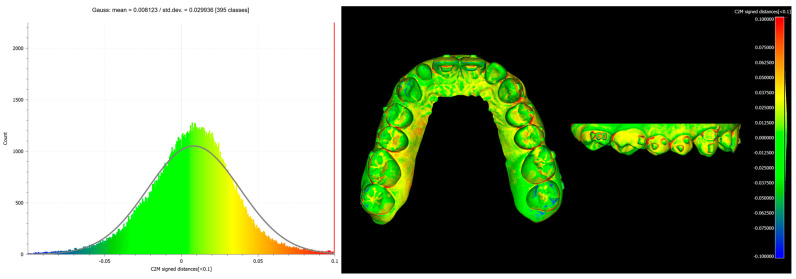
Color map and deviation histogram of the PrimeScan scanner showing trueness against the reference model.

To evaluate trueness, each scan was aligned to the reference Smart Optics model, and deviations were visualized using color maps and histograms. Representative color maps are shown in Figure 3, whereas the remaining visualizations are presented in the Supplementary Figures S1–S4. A ±0.1 mm scalar field range was applied to the color maps, where blue indicated inward deviations and red indicated outward deviations. Histograms were also generated using a ±0.1 mm range to visualize the distribution of deviations. These figures illustrate the trueness assessment by showing the deviation patterns between each intraoral scanner dataset and the reference model.

**Figure 4:**
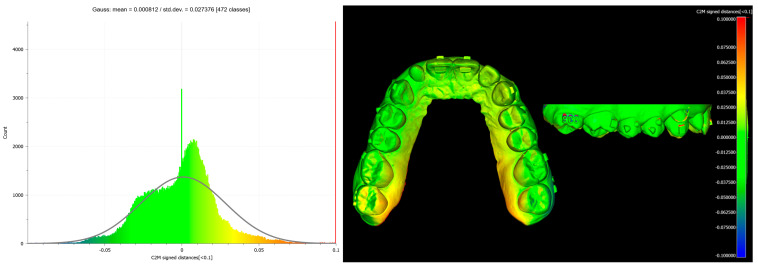
Color maps and histograms showing the deviation distribution of PrimeScan in precision analysis.

Deviations were examined using RMS values and after alignment and trimming, the models were imported into CloudCompare, color maps with histograms. Representative visualizations are shown in Figure 4, whereas additional color maps are available in the Supplementary Figures S5–S8. A ±0.1 mm range was applied to reflect how closely each scanner matched the reference. These figures specifically illustrate the precision assessment, demonstrating the consistency of repeated scans for each intraoral scanner.

**Figure 5:**
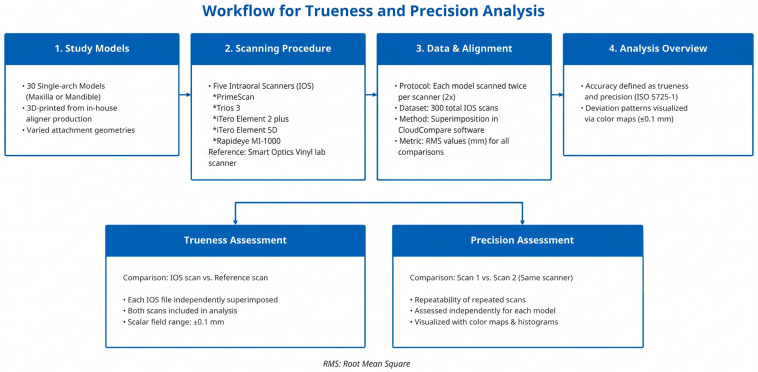
Study workflow for trueness and precision assessment of the evaluated intraoral scanners.

A schematic overview of the study design, scanning procedures, and trueness and precision assessment workflow is presented in Figure 5.

### 2.4. Statistical Analysis

Descriptive statistics including the mean, median, minimum, maximum, and interquartile range (IQR) were calculated. Since the assumptions of normality and homogeneity of variance were not met, non-parametric analyses were conducted. The Kruskal–Wallis test was applied to compare groups, and when significant differences were found, Dunn’s test with Bonferroni correction was used for pairwise multiple comparisons. All statistical analyses and graphical visualizations were performed using RStudio (version 2025.09.2, Posit Software, PBC).

(ICC) analysis was performed. The scanned models were re-aligned by the same operator after one month. The resulting ICC value was 0.987, indicating excellent agreement.

## 3. Results

To evaluate the scanning accuracy of five intraoral scanners, both precision (RMS deviation) and trueness (mean distance to reference) were analyzed. Complete descriptive statistics for precision and trueness are presented in Table 2 and Table 3, respectively. Since the data violated the assumptions of normality and homogeneity of variance, non-parametric Kruskal–Wallis tests were conducted. Statistically significant differences were found among the scanners for both precision (χ^2^ = 77.495, *p* < 0.001) and trueness (χ^2^ = 51.094, *p* < 0.001).

**Figure 6:**
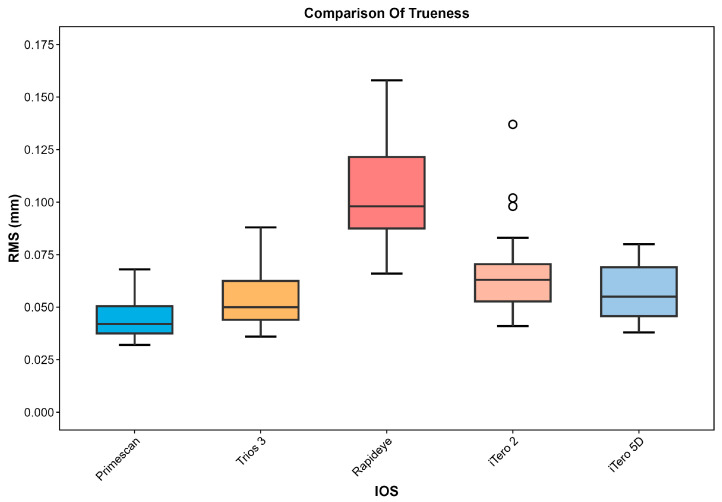
Boxplot comparison of trueness (RMS values) for the five intraoral scanners.

Regarding trueness, Primescan demonstrated the best performance, with significantly lower mean deviation values than Rapideye MI-1000 and iTero Element 2 plus (*p* < 0.01) and lower values than iTero element 5D, although this difference did not remain significant after Bonferroni correction (*p* = 0.086) (Table 4 and Figure 6). TRIOS 3 significantly outperformed Rapideye MI-1000 (*p* < 0.001), whereas no significant differences were observed between TRIOS 3 and iTero Element 2 plus (*p* = 0.663), TRIOS 3 and iTero element 5D (*p* = 1.000), or TRIOS 3 and Primescan (*p* = 0.720). Likewise, no significant difference was found between iTero Element 2 plus and iTero element 5D (*p* = 1.000). These findings suggest that Primescan, TRIOS 3, and iTero element 5D exhibited comparable trueness, while Rapideye MI-1000 demonstrated the lowest trueness performance among the evaluated scanners.

**Figure 7:**
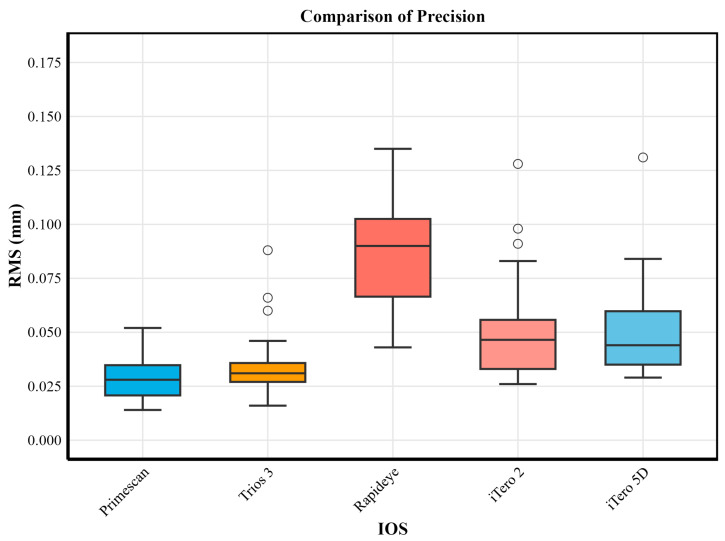
Boxplot comparison of precision (RMS values) for the five intraoral scanners.

For precision, Dunn’s post hoc tests with Bonferroni correction revealed that Primescan again performed best, showing significantly lower RMS values than Rapideye MI-1000, iTero Element 2 plus, and iTero element 5D (*p* < 0.001 for all) (Table 5 and Figure 7). TRIOS 3 also exhibited superior precision compared to Rapideye MI-1000 (*p* < 0.001), iTero Element 2 plus (*p* = 0.022), and iTero element 5D (*p* = 0.009). No significant difference was found between Primescan and TRIOS 3 (*p* = 1.000), nor between iTero Element 2 plus and iTero element 5D (*p* = 1.000). Rapideye MI-1000 had the poorest precision, differing significantly from all other scanners (*p* < 0.01).

## 4. Discussion

The null hypothesis of the present study, which stated that there would be no significant differences among intraoral scanners in terms of trueness and precision in 3D-printed orthodontic models with attachments, was rejected. Significant differences were observed among the evaluated scanners for both parameters.

In terms of trueness, there was no statistically significant difference among Primescan, 3Shape TRIOS 3, and iTero element 5D (*p* > 0.05). In contrast, the Rapideye MI-1000 scanner consistently exhibited the poorest performance, with significantly higher deviation values compared to all other scanners (*p* < 0.001), indicating lower trueness. Although iTero Plus 2 also demonstrated good performance, a significant difference was observed compared with Primescan (*p* < 0.001) (Table 4). A similar trend was observed when evaluating the scanners’ performance both through the STL images and the color-coded deviation maps (Figure 3 and Supplementary Figures S1–S4). Visual inspection of the STL meshes suggested a denser representation in Primescan scans, particularly in attachment regions. The accurate reproduction of small attachment geometries may have implications for digital orthodontic manufacturing workflows and warrants further investigation regarding its potential impact on aligner fabrication.

The trueness of the IOS was evaluated by comparing each digital model with the reference dataset obtained from the Smart Optics Vinyl laboratory scanner, which has a reported accuracy of approximately 6 µm [18]. In addition, similar Smart Optics laboratory scanners with reported accuracies of approximately 4 µm have been widely used as reference devices in previous studies evaluating intraoral scanner performance and digital model accuracy [19,20]. Nevertheless, unlike industrial metrology scanners or coordinate-measuring systems that are often regarded as gold standards, laboratory scanners may introduce a degree of systematic measurement bias that should be considered when interpreting the results.

When examining the color maps of scanners with lower deviation values, we noticed greater deviations in interproximal and undercut areas. We believe this may be due to the laboratory scanner’s limited ability to capture the model from all angles as effectively as intraoral scanners. Proper scanning of interproximal areas plays an important role in the accurate segmentation of teeth, which in turn allows for more optimal tooth movement planning and may positively affect overall treatment efficiency. In addition, the increasing use of artificial intelligence-based orthodontic software in recent years enables these systems to be trained with more accurate data derived from teeth and intraoral tissues [21,22]. In addition to scanner performance, the dimensional accuracy of digitally manufactured orthodontic appliances is also influenced by the behavior of printable dental biomaterials during photopolymerization and post-processing stages. Consequently, accurate digital acquisition of attachment geometries represents a critical prerequisite for maintaining dimensional fidelity throughout the entire additive manufacturing chain.

To compare the precision levels of the scanners, the obtained data were analyzed using a superimposition method. In multiple comparisons, the PrimeScan scanner demonstrated the highest precision and was found to be statistically significantly superior to iTero Element 2 plus (*p* = 0.001), iTero element 5D (*p* < 0.001), and Rapideye MI-1000 (*p* < 0.001). No significant difference was found between PrimeScan and TRIOS 3 (*p* = 1.000), and TRIOS 3 also showed high repeatability within its own group. TRIOS 3 was significantly superior to iTero Element 2 plus (*p* = 0.022), iTero element 5D (*p* = 0.009), and Rapideye (*p* < 0.001). No difference was observed between iTero Element 2 plus and iTero element 5D (*p* = 1.000); however, both devices performed significantly better than Rapideye MI-1000 (*p* = 0.001 and *p* = 0.002, respectively) (Table 5).

Scanner-related errors within approximately 50 μm are generally considered clinically acceptable for orthodontic diagnosis and treatment planning [23,24]. However, in aligner-based workflows, where high accuracy is needed to capture interproximal contacts and small features such as attachments, scanner performance becomes critical. In clear aligner therapy, some tooth movements per stage may be around 250 μm; thus, a 50 μm error would represent nearly 20% of this planned displacement. In addition, since errors may occur during the printing and thermoforming stages, we believe that the scanner accuracy should ideally be around 50 μm to minimize the cumulative effect of these deviations. The present findings should also be interpreted within the broader context of additive manufacturing-based orthodontic workflows. In contemporary in-office aligner production systems, inaccuracies originating during intraoral scanning may propagate throughout subsequent stages, including STL generation, 3D printing, and thermoforming procedures. Therefore, scanners capable of accurately reproducing attachment geometries and surface morphology may have implications for digital orthodontic manufacturing workflows. However, the potential effects of scanner accuracy on aligner adaptation, biomechanical force delivery, and clinical outcomes require further investigation.

Diker et al. [25], assessed the accuracy of commonly used scanners in daily clinical practice, including Trios 3, iTero element 2, Omnicam, Primescan, Virtuo Vivo, and Emerald. In results, the trueness and precision values were the lowest with the Primescan (25 and 10 μm), followed by Trios (40.5 and 11 μm), Omnicam (41.5 μm and 18 μm), Virtuo Vivo (52 and 37 μm), iTero (70 and 12 μm) and Emerald (73.5 and 60 μm). Primescan was identified as the scanner with the highest accuracy, while Emerald exhibited the lowest. Additionally, the accuracy of Virtuo Vivo was found to be statistically similar to that of Omnicam, Trios 3, and iTero element 2 [25].

A separate study by Ender et al. [26] compared the accuracy of various IOS and conventional impression methods across complete and partial arches. The tested devices included CEREC Omnicam, Primescan, TRIOS 3, iTero Element 2, Medit i500, and Planmeca Emerald. For complete-arch digital impressions, trueness values ranged from 16.3 ± 2.8 µm to 89.8 ± 26.1 µm, and precision values ranged from 10.6 ± 3.8 µm to 58.6 ± 38.4 µm, depending on the device. Although the scanning protocols and study models differed from ours, the general trend aligns with our findings, particularly regarding the superior performance of Primescan and TRIOS 3. In contrast to Ender et al.’s [26] study, which used conventional and full-arch typodont models, our study focused on more clinically complex anatomical conditions, including models with detailed structures such as attachments. Attachments, in particular, pose a greater challenge for intraoral scanners due to their small size and prominent edges. By incorporating such features, our study better reflects the high-resolution demands of in-office aligner workflows and emphasizes the importance of accurately capturing these anatomically complex regions.

Medina-Sotomayor et al. [27] explicitly reported that the Trios scanner exhibited superior accuracy compared to the iTero system. In their in vitro study, they assessed four intraoral scanners (Trios, iTero, Omnicam, and True Definition) using a high-precision industrial scanner as the reference standard. Multiple scanning strategies were employed, and each was repeated ten times on a complete maxillary arch cast. In this regard, the results of our study are consistent with those findings. In a study evaluating different scanners for single abutment impressions, TRIOS 3 was reported to have favorable trueness and precision values across both posterior and full-arch segments, particularly outperforming devices like Omnicam and TrueDefinition in various conditions [28].

On the other hand, some studies have suggested that the differences in trueness among intraoral scanner models are minimal and do not have a significant impact on routine clinical use [9,11,29]. Solaberrieta et al. [11], emphasized that ease of use and scanning speed are more important for clinicians than minor differences in scanner accuracy. Jacob et al. [15] reported that all scanners used in their study (iTero Element, Lythos, and Ortho Insight 3D) provided reliable and comparable measurements. Similarly, Latham et al. [30], compared full-arch scans using four different scanning strategies with Cerec Omnicam, Planmeca Emerald, iTero Element, and Trios, and found that iTero, Emerald, and Trios demonstrated similar accuracy. However, they noted that variations in accuracy could occur depending on the scanning strategy employed [30].

According to the literature, the accuracy of IOS is influenced by operator-related factors [31], environmental conditions such as temperature, light, and humidity [32], as well as the inherent characteristics of the scanner itself [26,30]. In the present study, all scans were performed by the same operator using calibrated devices under standardized environmental conditions. Thus, the only variable intentionally controlled and compared was the type of scanner used.

The models used in this study consisted of 30 different samples, each featuring various sizes of attachments and embedded text. These models were randomly selected from different patient groups undergoing in-house aligner production, distinguishing the present study from many previous investigations. Qualitative visual assessment of the scan images suggested that Primescan reproduced attachment geometries with a high level of detail, whereas numerical markings appeared to be more clearly represented by TRIOS 3, Primescan, and iTero element 5D scanners (Figure 1 and Figure 2).

In our study, the Smart Optics Vinyl laboratory scanner was selected as the reference device. Although industrial metrology scanners and coordinate-measuring systems are generally considered reference standards, such equipment was not available for this study. Therefore, the use of a dental laboratory scanner as the reference should be considered a limitation. Deviations were quantified using the widely accepted RMS metric, while the exclusion of other complementary accuracy parameters, such as the Hausdorff distance and mean absolute difference, constituted another methodological constraint. Furthermore, this investigation was conducted under in vitro conditions and did not replicate clinical factors such as saliva, soft tissue interference, patient movement, or limited intraoral access. All scans were performed by a single operator; therefore, inter-operator variability was not evaluated. In addition, scanner performance may be influenced by future software and firmware updates, and the present findings are limited to the software versions tested in this study. Since this was an in vitro study, scanning durations were not recorded. Although the models included attachments of varying geometries, no subgroup analyses were performed according to attachment size, shape, or location. Additionally, the use of models obtained from a single institution may limit the generalizability of the findings. Finally, although scanner accuracy was evaluated, downstream manufacturing outcomes such as 3D-printing accuracy, thermoformed aligner adaptation, and clinical treatment performance were not assessed. Future studies evaluating scanning time, additive manufacturing workflows, the effects of attachment characteristics, and the cumulative effects of scanning and 3D-printing deviations may provide further insights into the clinical feasibility of digital orthodontic workflows.

## 5. Conclusions

When overall scanning accuracy was considered by combining both trueness and precision outcomes, Primescan and TRIOS 3 demonstrated favorable overall performance among the evaluated intraoral scanners. Their ability to capture fine anatomical details with minimal deviation across scans highlights their suitability for additive manufacturing-based workflows, particularly in the fabrication of 3D-printed orthodontic models and in-office aligner production.

iTero Element 2 plus and iTero element 5D showed moderate performance, ranking below Primescan and TRIOS 3 in both trueness and precision, but still within a clinically acceptable range. Rapideye MI-1000, on the other hand, consistently demonstrated lower performance in both accuracy metrics, making it less suitable for the studied application.

## Figures and Tables

**Table 1:** Summary of the features and technical characteristics of the evaluated scanners.

IOS	Manufacturer	3D Optical Technology	Cost
PrimeScan	Dentsply Sirona, Charlotte, NC, USA	Confocal	~ $33.000
Trios 3	3Shape, Copenhagen, Denmark	Confocal	~ $25.000
iTero Element 2 plus	Align Technology, San Jose, CA, USA	Confocal	~ $17.800
iTero Element 5D	Align Technology, San Jose, CA, USA	Confocal	~ $15.600
Rapideye MI-1000	Mode Medical, Istanbul, Turkey	Triangulation	~ $7.200

Note: Prices are approximate and provided for general comparison purposes only. Actual market prices may vary according to region, distributor, and time of purchase.

**Table 2:** Descriptive statistics of RMS trueness values (mm) for the evaluated intraoral scanners.

Scanner	N	Mean	SD	Median	Minimum	Maximum	Q1	Q3
Primescan	30	0.045	0.011	0.042	0.032	0.068	0.037	0.050
Trios 3	30	0.054	0.014	0.050	0.036	0.088	0.044	0.062
iTero element 5D	30	0.057	0.013	0.055	0.038	0.080	0.046	0.069
iTero element 2 plus	30	0.066	0.022	0.063	0.041	0.137	0.053	0.071
Rapideye MI-1000	30	0.127	0.053	0.105	0.066	0.251	0.090	0.143

**Table 3:** Descriptive statistics of RMS precision values (mm) for the evaluated intraoral scanners.

Scanner	N	Mean	SD	Median	Minimum	Maximum	Q1	Q3
Primescan	30	0.0292	0.0098	0.0280	0.014	0.052	0.0207	0.0348
Trios 3	30	0.0341	0.0148	0.0310	0.016	0.088	0.0270	0.0357
iTero element 5D	30	0.0517	0.0235	0.0440	0.029	0.131	0.0350	0.0597
iTero element 2 plus	30	0.0514	0.0245	0.0465	0.026	0.128	0.0330	0.0558
Rapideye MI-1000	30	0.0866	0.0245	0.0900	0.043	0.135	0.0665	0.1025

**Table 4:** Pairwise comparisons of trueness among five intraoral scanners (RMS).

Comparison	Z Value	*p* Value	Adj. Sig.
Primescan—Trios 3	−1.799	0.720	0.720
Primescan—iTero element 5D	2.629	0.086	0.086
Primescan- iTero element 2 plus	3.636	0.003	0.003 **
Primescan—Rapideye MI-1000	−7.563	0.000	0.001 ***
Trios 3—iTero element 5D	0.830	1.000	1.000
Trios 3—iTero element 2 plus	1.837	0.663	0.663
Trios 3—Rapideye MI-1000	5.763	0.000	0.001 ***
iTero element 5D—iTero element 2 plus	1.007	1.000	1.000
iTero element 5D—Rapideye MI-1000	−4.933	0.000	0.000 ***
iTero element 2 plus—Rapideye MI-1000	−3.927	0.001	0.001 ***

*p* < 0.01 **, *p* < 0.001 ***, Dunn’s test with Bonferroni correction following a Kruskal–Wallis analysis.

**Table 5:** Pairwise comparisons of precision among five intraoral scanners (RMS).

Comparison	Z Value	*p* Value	Adj. Sig.
Primescan—Trios 3	0.868	0.385	1.000
Primescan—iTero element 5D	4.178	0.000	0.000 ***
Primescan—iTero element 2 plus	3.932	0.000	0.001 **
Primescan—Rapideye MI-1000	7.860	0.000	0.000 ***
Trios 3—iTero element 5D	3.310	0.001	0.009 **
Trios 3—iTero element 2 plus	3.064	0.002	0.022 *
Trios 3—Rapideye MI-1000	6.992	0.000	0.000 ***
iTero element 5D—iTero element 2 plus	0.245	0.806	1.000
iTero element 5D—Rapideye MI-1000	−3.683	0.000	0.002 **
iTero element 2 plus—Rapideye MI-1000	−3.928	0.000	0.001 **

*p* < 0.05 *, *p* < 0.01 **, *p* < 0.001 ***, Dunn’s test with Bonferroni correction following a Kruskal–Wallis analysis.

## Data Availability

The data presented in this study are available on request from the corresponding author. The data are not publicly available due to privacy and ethical restrictions related to participant data.

## Supplementary Materials

The following supporting information can be downloaded at: https://www.mdpi.com/article/10.3390/bioengineering13060709/s1, Figure S1: Color map and deviation histogram of the 3Shape TRIOS 3 scanner showing trueness against the reference model; Figure S2: Color map and deviation histogram of the iTero Element 5D scanner showing trueness against the reference model; Figure S3: Color map and deviation histogram of the iTero Element 2 plus scanner showing trueness against the reference model; Figure S4: Color map and deviation histogram of the Rapideye scanner showing trueness against the reference model; Figure S5: Color maps and histograms showing the deviation distribution of Trios 3 in precision analysis; Figure S6: Color maps and histograms showing the deviation distribution of iTero Element 5D in precision analysis; Figure S7: Color maps and histograms showing the deviation distribution of iTero Element 2 plus in precision analysis; Figure S8: Color maps and histograms showing the deviation distribution of Rapideye in precision analysis.

## Abbreviations

The following abbreviations are used in this manuscript:

IOS	Intraoral Scanner
CAD/CAM	Computer-Aided Design/Computer-Aided Manufacturing
3D	Three-Dimensional
RMS	Root Mean Square
ISO	International Organization for Standardization
