# Supplementary material for: Trueness and Precision of Intraoral Scanners for 3D-Printed Orthodontic Models with Attachments: An In Vitro Comparative Study
Source: Bioengineering (Basel). 2026 Jun 20;13(6):709. doi: 10.3390/bioengineering13060709 (PMC13295442; doi:10.3390/bioengineering13060709)
Supplement: Supplementary file 1 [file bioengineering-13-00709-s001.zip › bioengineering-4339870-supplementary.pdf]

Supplementary Figures

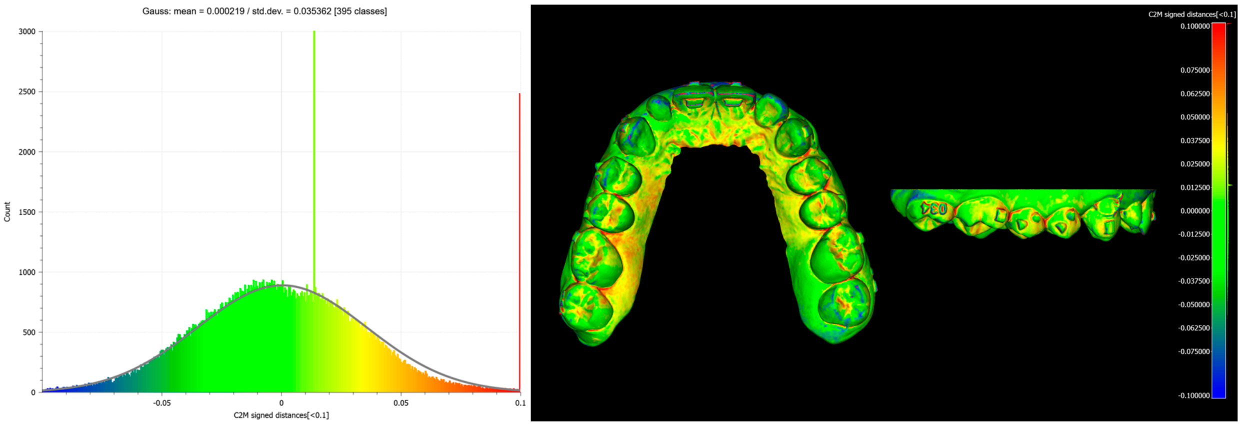

Supplementary Figure S1. Color map and deviation histogram of the 3Shape TRIOS 3 scanner showing trueness against the reference model.

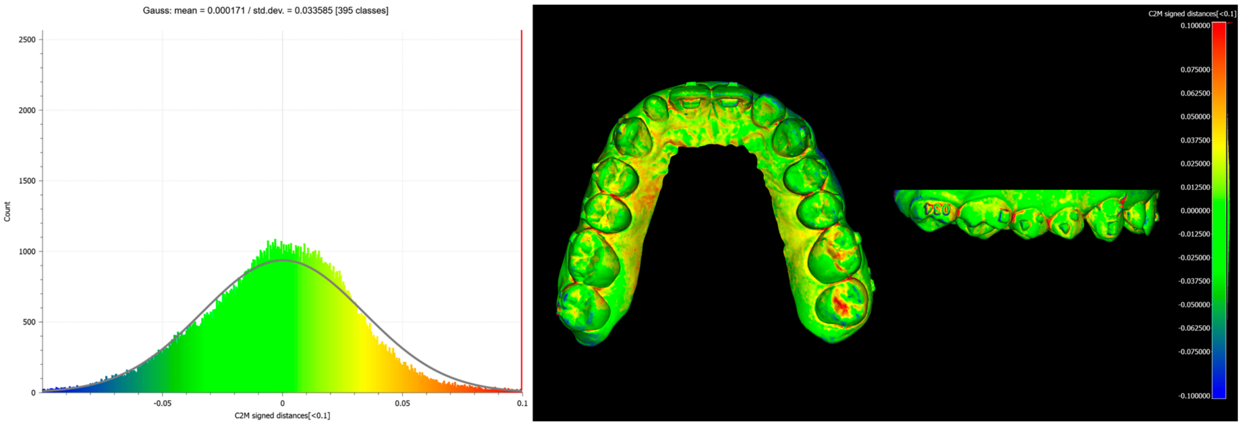

Supplementary Figure S2. Color map and deviation histogram of the iTero Element 5D scanner showing trueness against the reference model.

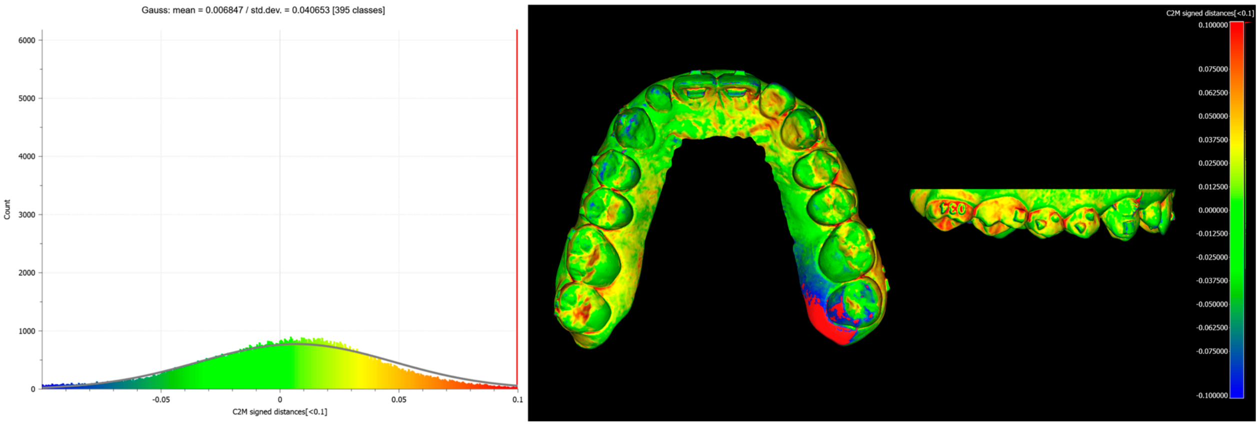

Supplementary Figure S3. Color map and deviation histogram of the iTero Element 2 plus scanner showing trueness against the reference model.

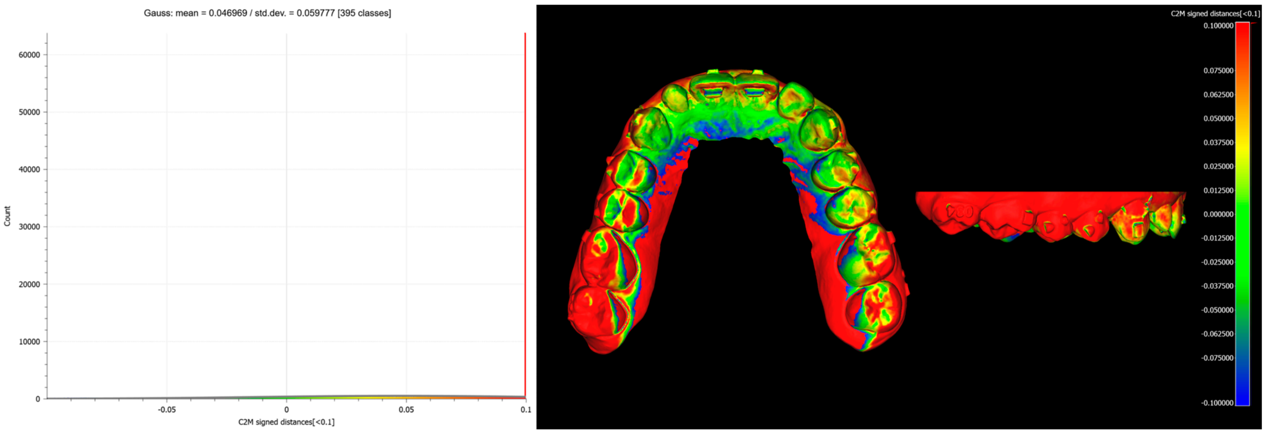

Supplementary Figure S4. Color map and deviation histogram of the Rapideye scanner showing trueness against the reference model.

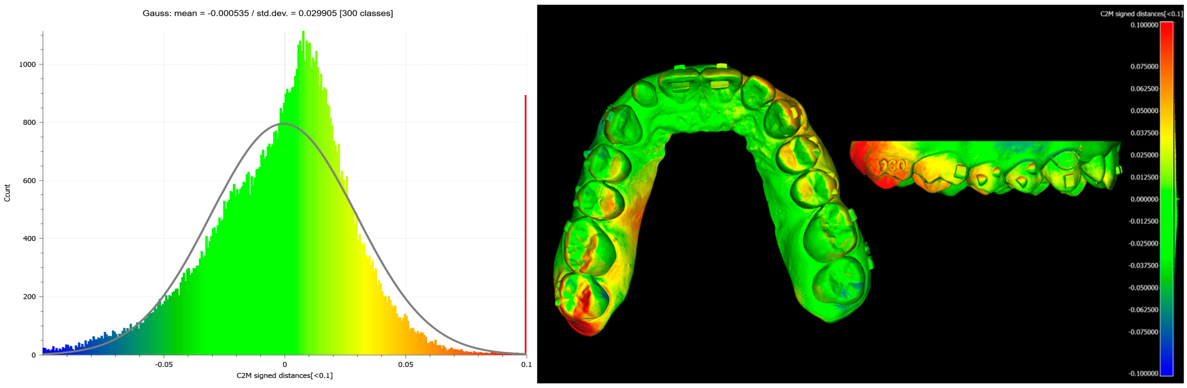

Supplementary Figure S5. Color maps and histograms showing the deviation distribution of Trios 3 in precision analysis.

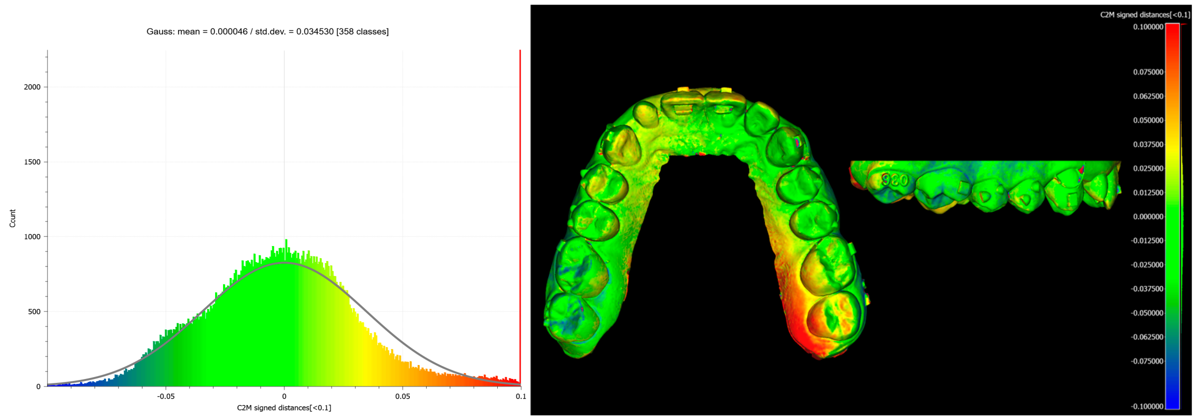

Supplementary Figure S6. Color maps and histograms showing the deviation distribution of iTero Element 5D in precision analysis.

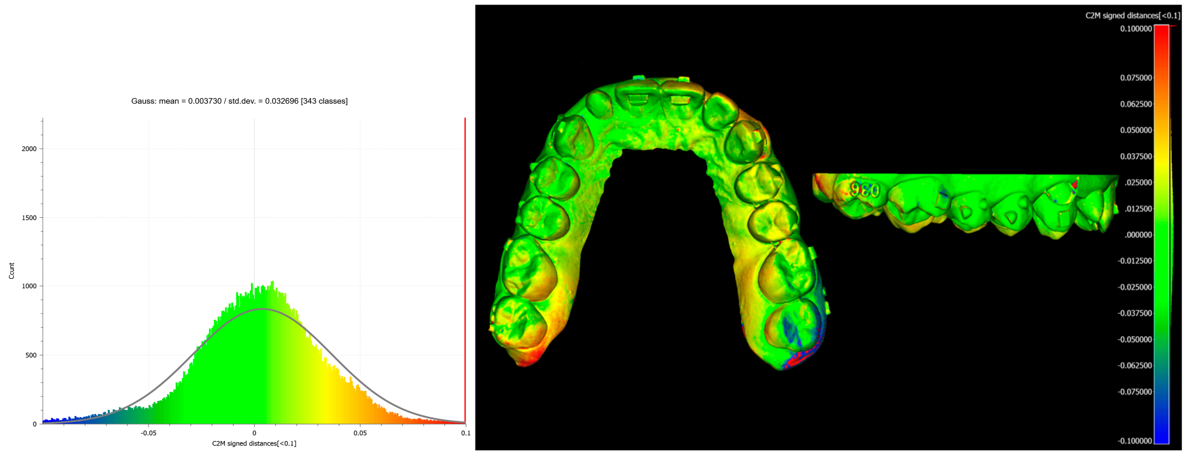

Supplementary Figure S7. Color maps and histograms showing the deviation distribution of iTero Element 2 plus in precision analysis.

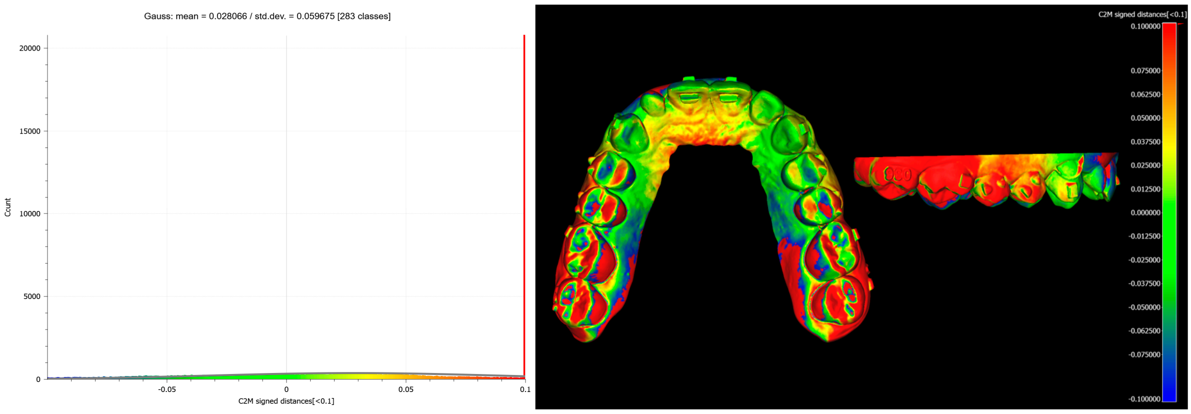

Supplementary Figure S8. Color maps and histograms showing the deviation distribution of Rapideye in precision analysis.
